# Population vitamin D status might be related to COVID-19 mortality but not with infection rate in Africa: evidence from ecological analysis

**DOI:** 10.11604/pamj.2022.41.249.29619

**Published:** 2022-03-25

**Authors:** Bereket Gebremichael, Demewoz Haile, Sibhatu Biadgilign

**Affiliations:** 1 College of Health Sciences, Addis Ababa University, Addis Ababa, Ethiopia,; 2 Public Health Nutrition Research Consultant, Addis Ababa, Ethiopia

**Keywords:** Vitamin D, COVID-19, mortality, infection, fatality, Africa

## Abstract

**Introduction:**

there is a large body of literature that has linked vitamin D status in the population with COVID-19 infection risk and disease severity. However, there is paucity of evidence in African context. Hence, this study aimed to conduct an ecological analysis to explore correlation between population level vitamin D status, COVID-19 infection, and mortality in Africa.

**Methods:**

an ecological study was conducted using data from different open sources, published literatures and organizational databases. In the final analysis, we included 23 African countries which had data on prevalence of vitamin D deficiency, population level mean serum 25 (OH) D concentrations and COVID-19 data. We employed spearman correlation and linear regression. All tests were two-sided, and P- value <0.05 was considered statistically significant.

**Results:**

based on our analysis, the prevalence of vitamin D deficiency is positively correlated (r=0.6265; p= 0.0094) while mean 25(OH) D concentration is negatively correlated (r=-0.4941; p= 0.0194) with COVID-19 mortality. In addition, the median age of the national population (r=0.7015; p= 0.0003), prevalence of current use of tobacco (r=0.6071; p= 0.0075) and prevalence of obesity among adult population (r=0.7143; p= 0.0003) were positively correlated with both COVID-19 infection and mortality in Africa. Nonetheless, vitamin D status was not positively correlated with observed case fatality rate and COVID-19 infection rate.

**Conclusion:**

population vitamin D status might be related to COVID-19 mortality but not with infection rate in Africa. Due to the increasing weight of evidence that shows a link between COVID-19 and vitamin D, we strongly recommend well-designed controlled studies to explore causality and clinical trials to find out the effect of vitamin-D supplementation in the treatment and prevention of COVID-19 in African settings.

## Introduction

The COVID-19 pandemic has presented one of the greatest challenges the world has faced in the history of critical care [1]. As of 30 January 2020, the World Health Organization (WHO) Director-General declared the novel coronavirus outbreak (2019-nCoV), later renamed to be COVID-19, as a Public Health Emergency of International Concern (PHEIC). COVID-19 is an acute viral illness, instigated by a new coronavirus called the SARS-CoV-2 (severe acute respiratory syndrome coronavirus) [2]. According to a WHO situational report, with the latest figure as of 29 March 2021, globally the cumulative number of COVID-19 confirmed cases reached 126,697,603 and 2,776,175 confirmed deaths since the start of the pandemic on 22 December, 2020 [3]. Originally, the SARSCoV-2 started in Wuhan, China, and has since spread around the world, as a pandemic and major global crisis [4]. The majority of COVID-19 infection and mortality were reported in high-income countries despite the presence of efficient health care systems in these countries [5]. The disease affects the entire health system through its direct effect as a communicable disease, as well as its ability to alter the overall mortality and burden of disease [4,6,7] which translated into a vast economic burden worldwide [7].

The majority of the individuals infected with the SARS-CoV-2 have mild to moderate respiratory disorders that resolve without pharmaceutical therapy. However, a considerable number of infected populations develop serious symptoms such as pneumonia and hypoxemia which may lead to death [8,9]. People with below-average vitamin D levels are more prone to serious COVID-19 symptoms and death [10,11]. Evidence also indicates that countries with high vitamin D deficiency prevalence are found to have high COVID-19 mortality [12,13]. Currently, there is a considerable interest to explore whether vitamin D might be used as adjuvant therapy in COVID-19 treatments. There are suggested biological mechanisms indicating that vitamin D could contribute to reduction of COVID-19 severity among infected patients [14]. An ecological study from Europe showed that countries with lower mean serum 5-hydroxyvitamin D [25(OH) D] concentration had higher odds of mortality from COVID-19 [15]. According to a recent systematic review published on The Lancet Global Health, vitamin D deficiency is common, affecting large segment of population in Africa [16]. This may predispose the African population to an increased risk of COVID-19 infection and its serious outcomes. However, there is no available research that has explored the link between vitamin D and COVID-19 in Africa. Therefore, this study aimed to conduct an ecological analysis to examine the correlation between the mean levels of vitamin D and vitamin D deficiency on COVID-19 infection and mortality in Africa.

## Methods

We followed the preferred method of reporting items for systematic reviews and meta-analyses (PRISMA) guidelines, for conducting the systematic review [17].

**Data source and search strategy:** we applied an ecological analysis using data extracted from the worldometers and published literature. Data on median age of the national population, global health security index, prevalence of current use tobacco and prevalence of obesity among adults are included in our analysis. The detailed description of the data abstraction procedure is explained in the following paragraphs.

**COVID-19 related data:** we obtained data on COVID-19 cases and mortality from the worldometers until January 15, 2021 [18]. We calculated observed case fatality rate and infection rate. Observed case fatality rate is defined as the proportion of people who die from COVID-19 among all individuals diagnosed with COVID-19 until January 15, 2020. For this analysis, the case fatality rate is presented per 10, 000 COVID-19 positive cases. Similarly, we defined infection rate as the proportion of people who were COVID-19 positive among susceptible population. In this study we assumed that everyone is susceptible, so we used the total population to calculate the infection rate. Similar to the case fatality rate, we presented the infection rate per 10,000 population. We calculated recovery ratio of COVID-19 by dividing the total number of recovered COVID-19 patients to total confirmed COVID-19 cases and multiply by 100.

**Vitamin D:** asystematic search was performed using PubMed, Scopus, Embase, Google Scholar, African Journals Online, and African Index Medicus, Cochrane Database of Systematic Reviews, Cochrane CENTRAL, and Web of Science to extract population level vitamin D status among adults in Africa, for papers without publication date, language, and text availability and article type restrictions. All the search terms were Medical Subject Heading terms, including vitamin D terms (“vitamin D”, “vitamin D deficiency”, “25-hydroxyvitamin D”, “calcifediol”, “ergocalciferols”, and “cholecalciferol”) and terms for African countries. Whenever we have two or more studies per country, we pooled the estimates together (prevalence weighted by sample size) to have a single pooled estimates of vitamin D status per country. In our review, we found that a total of 23 countries in Africa have vitamin D data. Mean vitamin D status was reported in all 23 countries and 17 of the countries have reported vitamin D deficiency data. Vitamin D deficiency was defined as having serum 25(OH) D concentration less than 30 nmol/L [16]. We included all studies that met the inclusion criteria and that had data available before January 10, 2021. We also manually screened citations of relevant articles to identify additional studies. The inclusion criteria were as follows: an original article published in a peer-reviewed journal; participants residing in Africa; observational studies (cross-sectional, longitudinal, case-control (control groups only), randomized clinical trial (baseline/placebo groups); study conducted on community or in hospital settings on apparently healthy population and the study measured 25(OH) D in blood. We excluded studies that were conducted outside Africa; were case reports and case series; studies with newborn babies, or pregnant women or new mothers; or only abstract or unpublished material available.

**Other variables:** we obtained data on median age of the national population in 2020 [19], global health security index [20], percentage who currently use tobacco [21] and prevalence of obesity among adults, BMI ≥30kg/m^2^ (%) (2016) [22] from other online sources and journals.

**Statistical analysis:** all analyses of the data were performed using R software package (version 3.6.0). We applied spearman correlation to examine if COVID-19 mortality, observed COVID-19 case fatality rate, COVID-19 infection rate and observed recovery rate were related with prevalence of obesity, tobacco use, median age of population and global health security index score. We applied linear regression to examine the association of vitamin D status (Vitamin D deficiency and mean serum vitamin D status) with COVID-19 mortality rate, observed COVID-19 case fatality, COVID-19 infection rate and observed COVID-19 recovery rate. We used scatter plots to visualize the correlations. All tests were two-sided, and a P value ˂ 0.05 was considered statistically significant.

**Ethical consideration:** this study does not require ethical approval by an ethics committee and all data are publicly available.

## Results

In this ecological analysis, we included 23 African countries (Nigeria, Ethiopia, Egypt, Democratic Republic of the Congo, Tanzania, South Africa, Kenya, Algeria, Sudan, Morocco, Uganda, Ghana, Ivory Coast, Cameroon, Malawi, Zimbabwe, Tunisia, Libya, Botswana, Gambia, Gabon, Guinea-Bissau and Seychelles) which had data on both prevalence of vitamin D deficiency or serum vitamin D level. However, from the above countries, 17 had data on prevalence of vitamin D deficiency. Democratic Republic of the Congo, Cameroon, Malawi, Zimbabwe, Gambia and Gabon had data on mean population vitamin D status but not on prevalence of vitamin D deficiency. Overall, approximately 34 persons out of 10, 000 population had a COVID-19 infection and 228 cases of observed COVID-19 resulted in fatality rate per 10,000 infections. Regarding vitamin D deficiency, the highest and lowest prevalence reported was 96.1% and 3.73% respectively. We summarized the COVID-19 related statistics and health related indicators among countries included in the study (Table 1).

**Table 1:** COVID-19 related statistics and health related indicators among countries included in the study (n= 22)

Variable	Mean	Std. Dev.	Min	Max
COVID-19 Infection rate per 10, 000 population	33.5	43.9	0.08	147.3
COVID-19 mortality rate per 10,000 population	0.69	1.09	0.003	3.99
Observed COVID-19 case-fatality rate per 10,000 infection	228.1	157.9	29.5	627.5
COVID-19 recovery rate (%)	9.6	9.50	3.6	31.8
Prevalence of vitamin D deficiency (%)	34.9	29.9	3.73	96.1
Mean 25(OH)D concentration(nmol/l)	68.5	19.7	24.2	110
Median age of the national population (years)	22.6	5.61	16.4	36.2
Global health security index	34.3	8.68	20	54.8
Prevalence of current use tobacco (%)	13.6	5.74	4.25	26.4
Prevalence of obesity among adults, BMI ≥30kg/m2 (%)	14.1	9.50	3.6	31.8

NB: Infection rate = # Infection*10,000/total population, Mortality rate = # death*10,000/total population, Observed case fatality ratio (CFR) = # death/ # COVID-19 confirmed cases *10,000,Recovery rates = # COVID-19 recovered/ # COVID-19 confirmed cases *100)

Based on our analysis, we found that COVID-19 mortality was positively correlated with prevalence of vitamin D deficiency (r=0.6265; p= 0.0094), while mean 25(OH) D concentration (r=-0.4941; p= 0.0194) was negatively statistically correlated with COVID-19 mortality. We did not find a significant correlation between vitamin D status and COVID-19 related infection (P-value> 0.05). However, median age of the national population (r=0.7438; p= 0.0000), prevalence of current use tobacco (r=0.5274 p= 0.0203) and prevalence of obesity among adults (r=0.6928; p= 0.0004) were positively and statistically correlated with COVID-19 infection. We also found median age of the national population (r=0.7015; p= 0.0003), prevalence of current use of tobacco (r=0.6071; p= 0.0075) and prevalence of obesity among adult population (r=0.7143; p= 0.0003) were positively correlated vitamin D mortality rate. Observed case fatality ratio was significantly correlated with the prevalence of current use of tobacco (r=0.4874; p= 0.0402). No other variable showed significant correlation with observed COVID-19 case fatality ratio including vitamin D status (Table 2).

**Table 2:** correlation of COVID-19 mortality rate, infection rate and observed COVID-19 case-fatality ratio with Vitamin D status and population level characteristics

Variable	Spearman Correlation Coefficient		
	COVID-19 Infection	COVID-19 mortality	COVID-19 observed case-fatality ratio
Prevalence of vitamin D deficiency	0.2843	0.6265*,**	0.2588
Mean 25(OH)D concentration	-0.3577	-0.4941*	-0.3077
Median age of the national population	0.7438*,**	0.7015*,**	0.0243
Global health security index	-0.1320	-0.1028	0.0040
Prevalence of current use tobacco	0.5274 *	0.6071*,**	0.4874 *
Prevalence of obesity among adults, BMI ≥30kg/m2 (%)	0.6928*,**	0.7143*,**	0.1429

* correlation is significant at the 0.05 level, **correlation is significant at the 0.01 level

**Figure 1:**
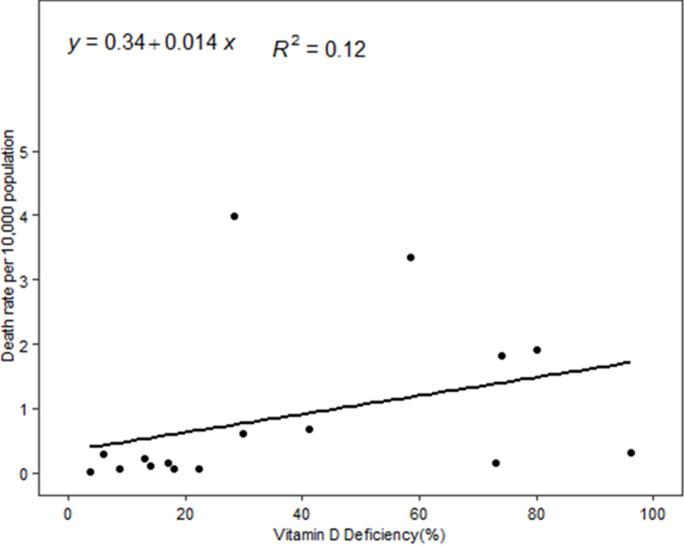
scatter plots between prevalence of vitamin D deficiency and COVID-19 mortality rate among 17 African countries

**Figure 2:**
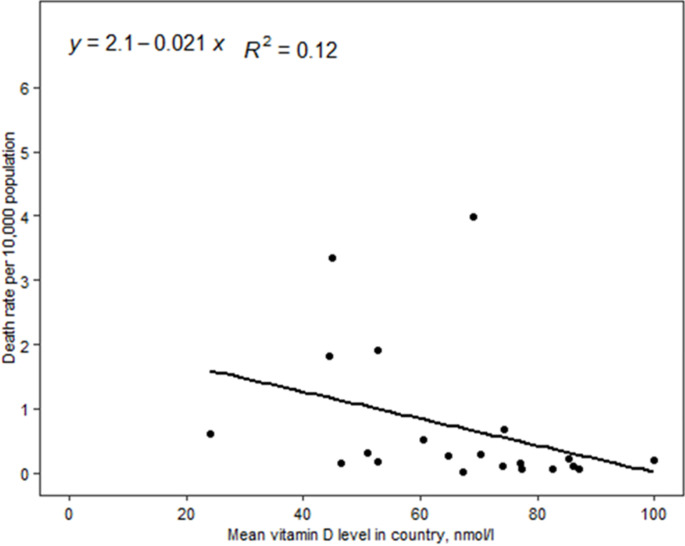
scatter plots between mean 25(OH) D concentration and COVID-19 mortality rate among 17 African countries

**Figure 3:**
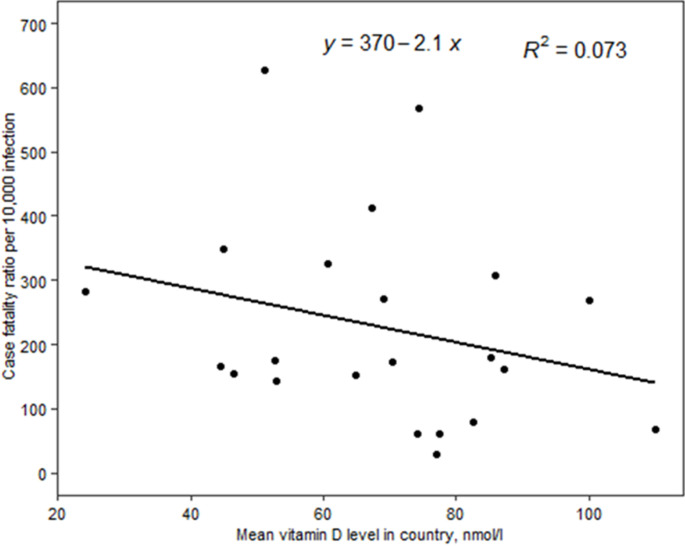
scatter plots between mean 25(OH) D concentration and observed case fatality rate among 17 African countries

**Figure 4:**
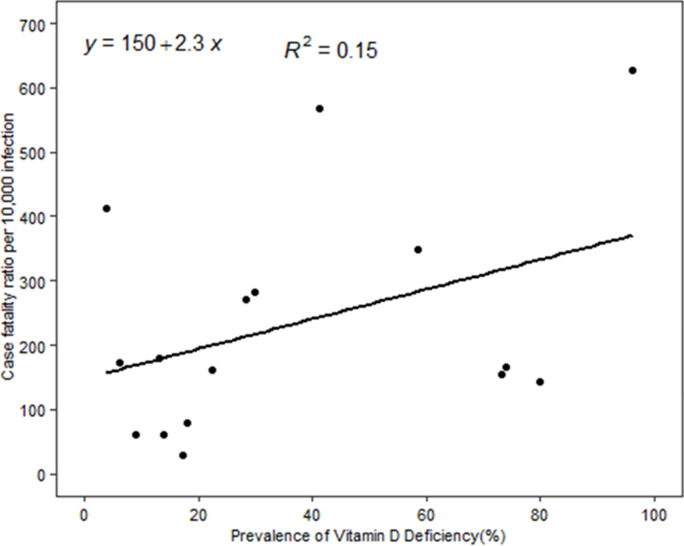
scatter plots between prevalence of Vitamin D deficiency and observed case fatality rate among 17 African countries

In the linear regression model, we did not find a statistically significant association between vitamin D deficiency and COVID-19 mortality. But the scatter plots showed a positive trend between COVID-19 mortality and vitamin D deficiency (Figure 1). Conversely, the scatter plots between mean 25(OH) D concentration and COVID-19 mortality showed a negative trend (Figure 2). We also employed linear regression to evaluate if vitamin D status is significantly associated with the observed case fatality rate. Our models did not show a significant association between vitamin D deficiencies and the observed case fatality rate, but the trend was positive. The scatter plots between mean 25 (OH) D concentrations and the observed COVID-19 case fatality rate was negative, but it was not a statistically significant association. Depicted in Figure 3, the scatter plots between mean 25 (OH) D concentrations in the country and observed case fatality rate showed a negative trend. As shown in Figure 4, the scatter plots showed a positive trend between observed case fatality rate and vitamin D deficiency.

## Discussion

Several recent studies have looked at the impact of vitamin D on COVID-19 [23-26]. This ecological study aimed to examine the correlation between the mean levels of vitamin D and vitamin D deficiency on COVID-19 infection and mortality in Africa. Based on our analysis, prevalence of vitamin D deficiency was positively correlated, while mean 25(OH) D concentration in a population was negatively correlated with COVID-19 mortality. In addition, prevalence of obesity in the adult population, median age of the population and current tobacco use were positively correlated with both COVID-19 infection and mortality in Africa. Early reports linked countries with lower mean vitamin D levels to higher mortality rates in the early course of the pandemic. Additionally, patients hospitalized with COVID-19 were found to have very low levels of 25-hydroxyvitamin D, providing evidence of this link. Currently, there are numerous studies that support the role of vitamin D in the risk and severity of COVID-19 infection [12,27-32]. Consistent with the current state of knowledge, our study found that the prevalence of vitamin D deficiency and mean 25(OH) D concentrations was positively and negatively correlated with COVID-19 mortality in Africa respectively. This relationship is biologically plausible as vitamin D affects both innate and adaptive immunity which in turn increases the antiviral potential of immune cells during respiratory viral infections, including COVID-19, and may reduce poor case outcomes [29,33-38]. In contrast, there are a number of reports that claim the link between Vitamin D and COVID-19 requires further investigation and is not yet conclusive despite seemingly strong evidence [31, 39,40]. Additionally, few studies claim the link is spurious [41,42]. Hence, further research is required to determine whether vitamin D and vitamin D deficiency have a role in prevention and treatment of COVID-19. Our linear regression models did not show a significant association of COVID-19 morality and observed case fatality rate with vitamin D deficiency and mean 25 (OH) D concentrations. This is most likely because the study was under powered as we included only 17 observations in our analysis.

An individual of any age may be infected by a virus. However, the human body's reaction to the virus depends greatly on the person's age. One of several key factors is that many older adults have a weakened immune system. In the context of the COVID-19 pandemic, numerous published reports have reported that many of the non-survivors are relatively elderly, which could be attributed to reduced immunity and co-morbidity in older patients [43-46]. Similarly, our study also revealed that median age of the national population is positively correlated with COVID-19 infection and mortality. However, recent reports have shown that in certain regions, the median age of new cases is declining; this could be due to increases in testing rates, which makes it possible to detect more mild or even asymptomatic cases, or other possible reasons requiring further assessment [47]. We therefore recommend an in-depth analysis of age as a risk factor to safeguard the older population in Africa by minimizing the spread of COVID-19. An increasing body of evidence suggested that obesity is strongly and independently correlated with poor COVID-19 outcomes, including death [48-52]. Our analysis also showed that prevalence of obesity among adult population is positively correlated to COVID-19 infection and mortality. There is a possible mechanism which may explain this relationship. Excess fat accumulation is linked to higher chronic subclinical inflammation, functional immunologic deficiency, and a pro-thrombotic condition, which may contribute to higher rates of disseminated intravascular coagulation and thromboembolism in severe COVID-19 patients [53].

In our study, African countries with a high proportion of tobacco use have a higher number of COVID-19 infections and mortality. Studies also showed that smoking doubles the mortality risk in covid-19[54-57]. On the contrary, another study demonstrated that smoking did not affect the severity of COVID-19 [58]. Even though smokers are more likely to develop acute respiratory distress syndrome and do worse with respiratory diseases [54], the impact of smoking on the transmission, severity and mortality of COVID-19 is not yet fully understood. Hence, it should be noted that the impact of current smoking on COVID-19 infection is a delicate and nuanced issue which should be thoroughly investigated before sending out potentially misinterpreted messages. The study should be interpreted with limitations. First, the measures of vitamin D status and COVID-19 cases are only a proxy based on the average in the population being sampled rather than individual level (ecological fallacy). Second, there could be a potential misclassification bias for the COVID-19 cases as the cases might differ from the number of true cases according to the definition of COVID-19 cases at the start of the pandemic and by country. Third, the completeness and consistency of the reporting of COVID-19 cases across countries creates another area of concern. Lastly, the COVID-19 pandemic is dynamic and rampant across the continent; the data used were the most recent available at the time of the analysis for this paper.

## Conclusion

We conclude that the population vitamin D status might be related to COVID-19 mortality but not with infection rate in Africa. Due to the increasing weight of evidence that shows a link between COVID-19 and vitamin D, we strongly recommend well-designed controlled studies to explore causality and clinical trials to determine the effect of vitamin-D supplementation in the treatment and prevention of COVID-19 in African settings.

### What is known about this topic

- The majority of COVID-19 infection and mortality were reported in high-income countries despite the presence of efficient health care systems;
- The disease affects the entire health system through its direct effect as a communicable disease, as well as its ability to alter the overall mortality and burden of disease;
- Evidences also indicate that countries with high vitamin D deficiency prevalence are found to have high COVID-19 death.

### What this study adds

- We found that COVID-19 mortality was positively correlated with prevalence of vitamin D deficiency while mean 25(OH) D concentration was negatively statistically correlated with COVID-19 mortality;
- We did not find a significant correlation between vitamin D status and COVID-19 related infection in our study;
- In addition, median age of the national population, prevalence of current use tobacco and prevalence of obesity among adults were positively and statistically correlated with COVID-19 infection and correlated vitamin D mortality rate.
